# Comparison of a full arch digital photographic assessment of caries prevalence in 5-year-old children to an established visual assessment method: a cross-sectional study

**DOI:** 10.1038/s41405-021-00087-0

**Published:** 2021-08-25

**Authors:** Nicole Thomas, Elizabeth Kay, Robert Witton, Cath Quinn

**Affiliations:** Faculty of Health, University of Plymouth, Plymouth, UK

**Keywords:** Dental epidemiology, Paediatric dentistry

## Abstract

**Introduction:**

Digital epidemiology in dental disease screening has a number of advantages which warrant further exploration.

**Aim:**

This study aimed to test the examination accuracy of digital images to evaluate child oral health by comparing the new method to a gold standard method. It also investigated the levels of diagnostic accuracy between different examiners, including dental care professionals and a lay examiner, when quantifying dental disease using images.

**Methods:**

A calibrated dental examiner inspected forty 5-year-olds. In addition, three sets of digital images were taken per child. These images were assessed by six examiners. Sensitivity and specificity of caries diagnosis and inter-examiner reliability were calculated to compare the caries scores derived from examination of the images to those of the gold standard examinations.

**Results:**

The mean values for sensitivity and specificity scores were 48.0% and 99.1%, respectively. The mean value for kappa showed moderate agreement between 0.43 and 0.73 (0.57). Mean values for agreement using intra-class coefficients were excellent (0.78) and good (0.73) for dt and dmft, respectively. No statistical difference in the validity of the caries scores was shown between the different image assessors.

**Conclusions:**

These data demonstrate the feasibility of using digital images to screen child oral health and for nondental professionals to be recruited to carry out digital epidemiology for the oral health surveillance of children.

## Introduction

Oral health epidemiology surveys first began in the UK in 1973 to determine the prevalence of dental disease in child and adult populations. Oral health survey findings are used to determine the extent of new oral health interventions and whether oral care provision should be maintained, expanded or reduced.^1^

Currently, there are a variety of dental caries assessment methods used in epidemiological studies to assess levels of dental disease in populations.^2–4^ In the UK, a well-documented clinical examination method developed by the British Association for the Study of Community Dentistry (BASCD) is used in national oral health surveys, and in child oral health dental research programmes, as the benchmark for population-level caries diagnosis. However, with changes in technology, the use of digital photography as a valid methodology for screening dental disease in a population has become a possibility.^5,6^ As a dental disease screening tool in epidemiology, or for data collection in oral health intervention research trials, it could offer considerable advantages over the standard visual examination. Benefit examples are, reducing resource costs, reducing the opportunity costs and guaranteeing the blinding of examinations. The advantages of digital technology for remote accessing and archiving have also been reported in the literature.^5–10^

In medicine and statistics, a gold standard (GS) test is usually the best available diagnostic test or benchmark, under reasonable conditions.^11^ In the few studies that have been published, the use of digital photography has been reported as equivalent to a benchmark GS method for the detection of caries.^8,9,12^ In addition, it has been shown to be acceptable to both examiners and young children.^13,14^ However, issues with food and debris obstructing diagnostic accuracy, and it being a time-intensive method, in comparison to the standard visual examination, have also been reported.^14^

Despite potential under reporting of disease, the BASCD screening method is the best system available likely to prevail in a school or community study, and is considered the caries surveillance method of choice.^15^ The BASCD method has also been the GS method of choice in previous digital photographic comparison studies.^7,9^

This study was designed to establish the feasibility and accuracy of using full arch digital images as a time-efficient method to screen dental disease in 4- to 5-year-old children, using the BASCD visual examination method as the GS. In addition, this paper reports the diagnostic accuracy of six independent examiners using the digital images, including five untrained dental care professionals and a lay person (LP). The diagnostic accuracy of an untrained dental examiner has not previously been explored.

The concurrent validity of the digital method was tested by calculating and comparing the sensitivity and specificity of caries diagnosis, and examining the inter-examiner reliability of the photographic assessors compared to a GS, calibrated BASCD examiner. The acceptability of the method along with comparing the results of this study with known caries prevalence rates was not included in the research design

## Methods

Ethical and regulatory approval was obtained for the study from the University of Plymouth Faculty Research Ethics Integrity Committee (FREIC) for Health and Human Sciences (17/18-863).

This was a cross-sectional study comparing the already established visual examination method developed by the BASCD with a digital photographic assessment method in a sample of 5-year-old children.

### Study population and recruitment

The population examined were reception children attending two schools in areas identified as high dental need in Plymouth, in South West England. The schools were identified by the high-uptake of free school meals that corresponded with the high rates of dental extractions under GA as reported by the Office of the Director of Public Health, Plymouth City Council.^16^ The purpose of using a child population known to have high caries rates was to guarantee the presence of caries for the digital images, therefore no demographic information was collected.

Prior to data collection, study invitation letters, study information sheets and consent forms were sent to parents/legal guardians of eligible children via the schools, informing them of the study. Completed consent forms were returned to the principal investigator (PI) on the morning of data collection. All participating children received a toothbrushing lesson using the dry brushing protocol,^17^ and a goody bag containing a toothbrush and toothpaste. Each child recruited into the study was assigned a unique identification number (ID).

### Examination and assessment

The examiner had been trained and calibrated to the BASCD caries examination protocol by members of the UK National Epidemiological Surveys team.^18^ Caries was diagnosed visually at the ‘caries into dentine’ level. Decayed, missing, filled teeth (dmft) scores were obtained from the data using the BASCD guidance on the statistical aspects of training and calibration of examiners for surveys of child dental health.^19^

The children had a visual dental examination according to the BASCD diagnostic protocol.^15^ Three digital views were then taken of their primary dentition: full upper arch, full lower arch and an anterior view.

Children were seen in groups of three to five. Toothbrushing instruction was given first by the PI (a qualified dental hygienist) to ensure teeth were clean and free from debris prior to the visual and digital examinations. The PI acted as assistant to the BASCD examiner, recording scores according to the BASCD criteria^15^ for each child onto a paper form marked with the child’s unique ID. All recommended instrumentation and equipment were used, as recommended in the BASCD protocol.^20^

From this point forward, the BASCD examiner will be referred to as the GS.

### Photographic procedures and assessments

Prior to taking the digital photographs, electronic folders were created in the data imaging software using the same unique IDs to those assigned to each participant. This was to ensure matching of the correct visual examination scores with the photographic scores. Data imaging software was installed onto a tablet (Surface Pro 4). Using a CS1500 (Carestream Dental^TM^) intraoral camera with integral light source, with images being taken by the PI. Children sat upright on the dental chair and two disposable mirrors were used to retract the oral tissues. Images were repeated until the PI was satisfied the best images possible had been captured, or until the child became less compliant with the procedure. These were automatically saved into the corresponding unique ID folder. Three images for each child were chosen and added to a bespoke document with the same BASCD criteria used for the visual examination method (see Supplementary material).

Files for each photographic assessor were saved onto a USB flash drive. One of the photographic assessors was a BASCD calibrated examiner (BCE), separate from the GS. The GS examiner did not assess the images. The remaining assessors were the PI, a general dental practitioner (GDP), a dental therapist (DT), a dental nurse (DN) and a lay person (LP). Apart from the BCE, the assessors had no formal training on how to assess caries from digital images. Deciduous central and lateral incisors that had naturally exfoliated were pre-scored as ‘missing’. Assessors were given the choice to use the score ‘9’, ordinarily used for when an assessment plaque score cannot be made,^20^ if they felt a judgement could not be made to a tooth surface. This was an arbitrary number for the purpose of recording no-scores for this study only. Each assessor was asked to give feedback on the time taken and any challenges they encountered.

The PI assessed the photographs up to 6 months later (after data collection and completion by the other digital assessors) to reduce observational bias.

### Data processing and analysis

All data were collated into IBM SPSS Statistics 24. Kappa statistic for agreement with the GS were generated using a dichotomous scoring of ‘sound’ or ‘unsound’ per tooth (*n* = 788). Due to the use of ‘assessment cannot be made’ on certain tooth surfaces, three separate rules were applied to the dichotomous scoring in order to compare the assessments (see Table 1).

**Table 1:** Sensitivity, specificity and kappa scores for each rule to compare photographic assessments to the gold standard.

	Rule 1: teeth with an ‘assessment cannot be made’ score removed from analysis				Rule 2: only teeth with all surfaces scored as ‘assessment cannot be made’ removed from analysis				Rule 3: only teeth with surfaces most likely to be affected by caries (8) and scored ‘assessment cannot be made’ removed from analysis			
	*N*	Sens %	Spec %	Kappa	*N*	Sens %	Spec %	Kappa	*N*	Sens %	Spec %	Kappa
PI	786	62.3	99.7	0.74	788	62.3	99.7	0.74	787	62.3	99.7	0.74
BCE	621	55.3	99.3	0.66	713	53.1	98.9	0.61	693	53.1	98.9	0.62
GDP	299	33.3	98.9	0.43	785	50.9	98.5	0.57	716	54.2	98.5	0.60
DT	765	47.2	98.5	0.54	788	47.2	98.4	0.53	783	47.2	98.4	0.53
DN	786	36.5	99.7	0.50	788	35.8	99.7	0.49	787	35.8	99.7	0.49
LP	788	41.5	99.2	0.52	788	41.5	99.2	0.52	788	41.5	99.2	0.52

Using the BASCD guidance on the statistical aspects of training and calibration of examiners for surveys of child dental health, dt and dmft scores were generated and analysed.^19^ Because of the small participant sample, mt and ft were not individually analysed as only two children presented with filled teeth or teeth extracted due to caries. Agreement between the GS and photographic assessors for dt and dmft was analysed using an intraclass correlation coefficient (ICC) matrix.

Bland–Altman plots were created to identify the limits of agreement (LOA) and to identify cases that fell outside of the 95% LOA. Cases outside the LOA were studied to determine possible reasons behind the lack of reliability.

Independent samples *T*-tests were used to explore any statistical difference between the GS mean and the photographic assessor mean scores, which may infer poor agreement.

## Results

A total of 43 children took part in the study. Of these, three children refused to have the visual examination and digital photographs. A total of 294 images were taken, an average of seven images per child. Taking the full arch digital images took up to 2 minutes per child. All photographs used for assessment (*n* = 120) were scored.

The sensitivity scores ranged between 32.7% (DN) and 62.3% (PI), with a mean value of 48.0%. These fall below the BASCD recommended 75% level as outlined in BASCD guidance on the statistical aspects of training and calibration of examiners for surveys of child dental health.^14^ Specificity scores ranged between 98.1% (BCE) and 99.6% (PI) with a mean value of 99.0%. These are above the BASCD recommended 90% level. The kappa statistics ranged between 0.43 and 0.73, with a mean of 0.57, showing moderate to substantial agreement^21^ between the photographic assessors and the GS. However, this falls below the 0.75 benchmark recommended by BASCD^19^ (see Table 1).

The ICC, as a measure of inter-examiner reliability, showed good agreement^22^ between most of the photographic assessors and GS dt scores (PI 0.85, GDP 0.88, DT 0.80, LP 0.82), and moderate agreement between the GS and BCE (0.63) and DN (0.71) scores. The mean ICC value for dt was 0.78 (good agreement). The PI and BCE showed good agreement with the GS (PI 0.89, BCE 0.77) for dmft scores, with the remaining photographic assessors showing good agreement (GDP 0.72, DT 0.70, DN 0.61, LP 0.71). The mean ICC value for dmft was 0.73.

**Fig. 1:**
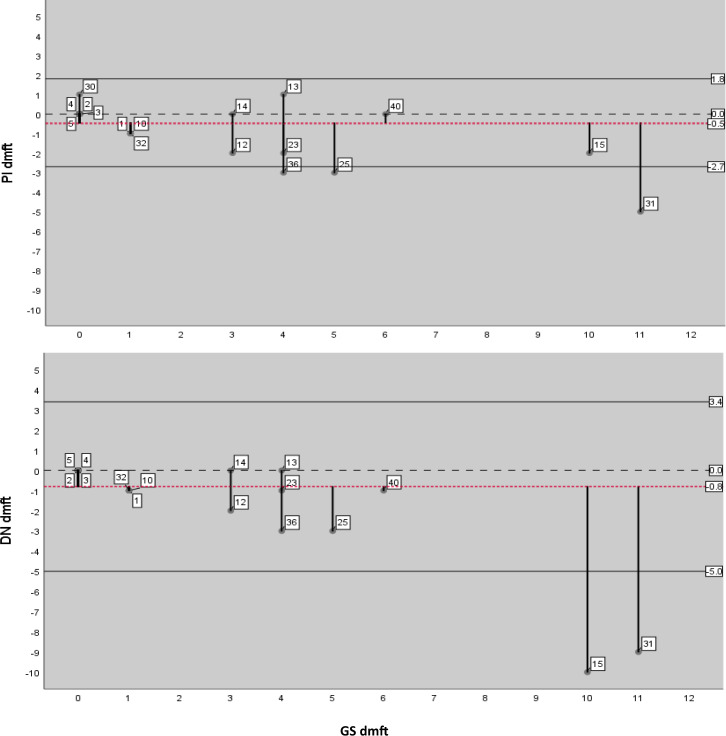
Bland-Altman plots showing the image assessors with the most (PI) and least (DN) agreement with the GS for dmft. Plots show 95% confidence intervals, means and divergent cases, in particular, case 15 and 31.

The 95% LOA comparing the dt photographic assessment scores with the GS were –2.6 to 1.8 (PI), –3.8 to 2.8 (BCE), –2.2 to 1.8 (GDP), –2.8 to 2.5 (DT), –3.6 to 2.6 (DN) and –2.8 to 2.1 (LP). The 95% LOA comparing the dmft photographic assessment scores with the GS were –2.7 to 1.8 (PI), –3.9 to 2.9 (BCE), –4.0 to 3.3 (GDP), –4.2 to 3.4 (DT), –5.0 to 3.4 (DN) and –4.4 to 3.1 (LP). There were seven sets of dental images that showed divergent results, with two cases consistently falling outside of the 95% LOA. These were identified by generating Bland–Altman graphs to aid visualisation of the LOA between assessors (see Fig. 1 for Bland–Altman plots showing the assessors with the most and least dmft agreement compared to the GS, and cases outside LOA).

Independent samples *T*-tests showed that all photographic assessors’ mean dt and dmft scores fell below the GS. No statistical difference was seen between photographic assessors compared to the GS. However, the DN dmft score showed an almost significant difference with *P* ≤ 0.09 (see Table 2).

**Table 2:** Results of independent samples T-Test from dt and dmft data with standard deviations and 95% confidence intervals.

	Mean (dt)	SD (dt)	Mean diff	*P* < 0.05 vs GS (dt)	95% confidence interval		Mean (dmft)	SD (dmft)	Mean diff	*P* < 0.05 vs GS (dmft)	95% confidence interval	
GS	1.05	2.17					1.33	2.71				
PI	0.65	1.50	0.4	0.34	–0.4	1.2	0.88	1.96	0.5	0.40	–0.6	1.5
BCE	0.58	1.36	0.5	0.24	–0.3	1.3	0.83	1.85	0.5	0.34	–0.5	1.5
GDP	0.85	1.99	0.2	0.67	–0.7	1.1	0.95	2.08	0.4	0.49	–0.7	1.4
HYG	0.88	1.40	0.2	0.67	–0.6	1.0	0.93	1.54	0.4	0.42	–0.6	1.4
DN	0.53	1.22	0.5	0.19	–0.3	1.3	0.53	1.22	0.8	0.09	–0.1	1.7
LP	0.70	1.62	0.4	0.42	–0.5	1.2	0.70	1.62	0.6	0.21	–0.4	1.6

## Discussion

This study used a novel approach for assessing the diagnostic accuracy of digital photographs by using six independent examiners, including a lay person. The aim was to demonstrate the versatility and validity of the photographic method as it can be used by both dental health professionals and non-dental professionals, therefore, no formal training on assessing caries on digital images was given. If lay examiners could be used in mass screening programmes this would greatly reduce the resource implications of large scale and national studies. A further advantage in the use of digital photographs and remote assessment would be that true blinding in research trials of dental interventions could be achieved. In addition, digital photographs could be accessed remotely and a digital archive can be used to track population changes over time thus facilitating longitudinal studies, which are currently a rarity due to the expense of repeated examination by professionals. This study also offers insights and solutions to some of the issues found in previous studies of digital examinations relating to time and image quality.^7,9^

Probably the most important finding in this study was the lay photographic assessment scores. No statistical difference was found between dt (*P* = 0.42; 95% CI [–0.5 to 1.2]) and dmft (*P* = 0.21; 95% CI [–0.4 to 1.6]) lay and GS scores. The lay inter-examiner showed similar, if not better reliability, in comparison to the other digital image examiners. This clearly indicates the feasibility for non-dental professionals to be recruited to carry out digital epidemiology for the oral health surveillance of children.

Oral health surveys are resource intensive. Sensitivity to workload is needed when scheduling, as inaccuracies and inconsistencies are more likely when the examiner is fatigued.^1^ A single observer is used for each region and although every care is taken to ensure calibration of each examiner, digital epidemiology would reduce opportunity costs by allowing multiple examiners to assess the images. The opportunity costs for the multiple children needing to be re-examined for intra-rater reliability would also be reduced.

Alongside the usual advantages to digital archiving for training purposes^15^ and contemporaneous record keeping, having a digital record from epidemiological surveys could also provide an opportunity to retrospectively extract further data from a digital database.^6^ Creating a digital archive for open source data would allow single databases to be used more widely, with data being leveraged, shared and combined with other data.^23^ Sharing of information in this way assists scientific collaboration, enriches research and advances analytical capacity to inform decisions.^23^

All photographic assessors were above the BASCD specificity benchmark (90%) for correctly recognising teeth free from dental disease (99.1%). However, all photographic assessors fell below the BASCD sensitivity benchmark (75%) for correctly recognising dental disease (57%). The PI was the closest to reaching the BASCD kappa benchmark (0.75) with a mean of 0.74, however, all other photographic assessors fell below this value. Similar underestimation of disease has been a consistent finding in almost all previous studies.^7,9^

Causes of underestimation of dental disease in this and previous studies were due to tooth coloured fillings being more difficult to identify, and transcription errors—when ‘extracted caries’ and ‘missing’ were interchanged.^14^ In this study, the angulation of the images was also problematic. The rationale for using full arch images with a single anterior view was to address the issues of time reported in the previous studies^7,9,14^ and to reduce additional resources needed for cross infection control. Boye et al. (2013) reported that taking digital images of all tooth surfaces took an average 8 min per child. Using a full arch image method reduced this time to a maximum of 2 min per child. However, this did affect the quality and diagnostic accuracy of the images.

More advanced technologies are becoming available, including handheld, intraoral high definition video devices. To date, the use of full mouth video technology for epidemiology is unexplored. Research in this area should be considered to test the validity of these modalities to address the underestimation of disease found in this and previous studies.^6^ Underestimation of disease can also be resolved by applying a correction factor to minimise bias.^24^ In addition, a possible area of interest may be including a comprehensive cost analysis when using digital images to screen for caries compared to a validated conventional method.

Currently, it is impossible to blind examiners for data collection in dental epidemiological studies, by the very nature of disease detection.^6^ Attempts to address issues such as participant identifiers or the examiner’s conscious or unconscious evaluation of a subject’s accent, vocabulary, dress or mannerism have been made.^6,25^ Inadequate blinding can cause significant differences in treatment effect size estimates.^26–28^ In well-designed research trials, possibility of bias is recognised and minimised as much as possible to ensure the integrity of the results. However, in disease measurement and reporting, blinding cannot be guaranteed. Digital dental disease screening as a data collection method could strengthen the blinding process.^5^

In this study, the photographic assessors were independent of the visual examination process, except for the PI. This meant that observation bias was minimised. The PI left a time gap of up to 6 months between assessing the digital photographs from both schools. Despite this, the PI reported a difficultly in remaining objective when scoring the photographs. This bias may be reflected in the results, where the PI showed consistently higher agreement with the GS in comparison the independent photographic assessors (see Tables 1 and 2).

Despite meeting the sample size reliability criteria,^22^ the sample size in this was considerably less than those tested in by Boye et al. (2013). Intra-rater reliability was not calculated due to the small sample size. A further limitation to this study was the use of one calibrated examiner for the visual examinations and one assessor from each category scoring the images. A combination of multiple visual examiners and multiple independent assessors would eliminate observational bias and test intra-examiner reliability.

## Conclusion

The findings of this study suggest that the use of digital images in dentistry shows continued promise as both an oral health screening tool and a research methodology. Although digital images may produce a level of underestimation of dental disease, for comparative studies they show great potential.^7,9^

Using digital images as a dental disease surveillance method significantly reduces resource and opportunity costs, and also has advantages relating to remote accessing, archiving and guaranteeing blinding for both epidemiological surveys and research trials. With the additional feasibility of using trained non-dental professionals to assess the digital images, the costs of both dental research and dental epidemiology could be dramatically reduced.

## Supplementary information

Supplementary information

## References

[1] World Health Organisation. (2013). Oral health surveys: basic methods.

[2] Ismail AI. Visual and visuo-tactile detection of dental caries. J Dent Res. 2004;83 Spec No C:C56–66. 10.1177/154405910408301s12 15286124

[3] Kuhnisch J (2009). Development, methodology and potential of the new Universal Visual Scoring System (UniViSS) for caries detection and diagnosis. Int J Environ Res Public Health.

[4] Jablonski-Momeni A (2008). Reproducibility and accuracy of the ICDAS-II for detection of occlusal caries in vitro. Caries Res.

[5] Forgie AH, Pine CM, Pitts NB (2003). The assessment of an intra-oral video camera as an aid to occlusal caries detection. Int Dent J.

[6] Hogan R (2018). Further opportunities for digital imaging in dental epidemiology. J Dent.

[7] Boye U (2013). Comparison of caries detection methods using varying numbers of intra-oral digital photographs with visual examination for epidemiology in children. BMC Oral Health.

[8] Boye U (2012). Comparison of photographic and visual assessment of occlusal caries with histology as the reference standard. BMC Oral Health.

[9] Boye U (2013). Comparison of an intra-oral photographic caries assessment with an established visual caries assessment method for use in dental epidemiological studies of children. Community Dent Oral Epidemiol.

[10] Estai M (2016). Diagnostic accuracy of teledentistry in the detection of dental caries: a systematic review. J Evid Based Dent Pract.

[11] Versi E (1992). “Gold standard” is an appropriate term. BMJ.

[12] Bottenberg P, et al. Comparison of occlusal caries detection using the ICDAS criteria on extracted teeth or their photographs. BMC Oral Health. 2016;16:93. 10.1186/s12903-016-0291-z PMC5015202 27604238

[13] Boye U (2012). Children’s views on the experience of a visual examination and intra-oral photographs to detect dental caries in epidemiological studies. Community Dent Health.

[14] Boye U (2013). The views of examiners on the use of intra-oral photographs to detect dental caries in epidemiological studies. Community Dent Health.

[15] Pitts NB, Evans DJ, Pine CM (1997). British Association for the Study of Community Dentistry (BASCD) diagnostic criteria for caries prevalence surveys-1996/97. Community Dent Health.

[16] Plymouth City Council. (2019). Dental extractions under general anaesthetic in Plymouth children.

[17] Public Health England. Improving oral health: a toolkit to support commissioning of supervised toothbrushing programmes in early years and school settings. 2016. https://www.gov.uk/government/publications/improving-oral-health-supervised-tooth-brushing-programme-toolkit. Accessed May 2016.

[18] Mitropoulos CM, Lennon MA, Worthington HV (1990). A national calibration exercise for the British Association for the Study of Community Dentistry regional examiners. Community Dent Health.

[19] Pine C, Pitts N, Nugent ZJ (1997). British Association for the Study of Community Dentistry (BASCD) guidance on the statistical aspects of training and calibration of examiners for surveys of child dental health. A BASCD coordinated dental epidemiology programme quality standard. Community Dent Health.

[20] Mitropoulos CM, Pitts NB, Deery C. BASCD trainers’ pack on caries diagnosis. Dundee: Dental Health Services Research Unit; 1992.

[21] Landis JR, Koch G (1977). The measurement of observer agreement for categorical data. Biometrics.

[22] Koo TK, Li MY (2016). A guideline of selecting and reporting intraclass correlation coefficients for reliability research. J Chiropr Med.

[23] Huston P, Edge VL, Bernier E (2019). Reaping the benefits of open data in public health. Can Commun Dis Rep.

[24] Kirkwood BR, Sterne JAC. Essential medical statistics. 2nd ed. Malden, MA: Blackwell Science; 2003.

[25] Stephen KW (2002). A blind caries and fluorosis prevalence study of school-children in naturally fluoridated and nonfluoridated townships of Morayshire, Scotland. Community Dent Oral Epidemiol.

[26] Saltaji H (2018). Influence of blinding on treatment effect size estimate in randomized controlled trials of oral health interventions. BMC Med Res Methodol.

[27] Karanicolas PJ, Farrokhyar F, Bhandari M (2010). Practical tips for surgical research: blinding: who, what, when, why, how?. Can J Surg.

[28] Fleming PS, Lynch CD, Pandis N (2014). Randomized controlled trials in dentistry: common pitfalls and how to avoid them. J Dent.

