# Supplementary figures and images for: Comparison of a full arch digital photographic assessment of caries prevalence in 5-year-old children to an established visual assessment method: a cross-sectional study
Source: BDJ Open. 2021 Aug 25;7:32. doi: 10.1038/s41405-021-00087-0 (PMC8387399; doi:10.1038/s41405-021-00087-0)

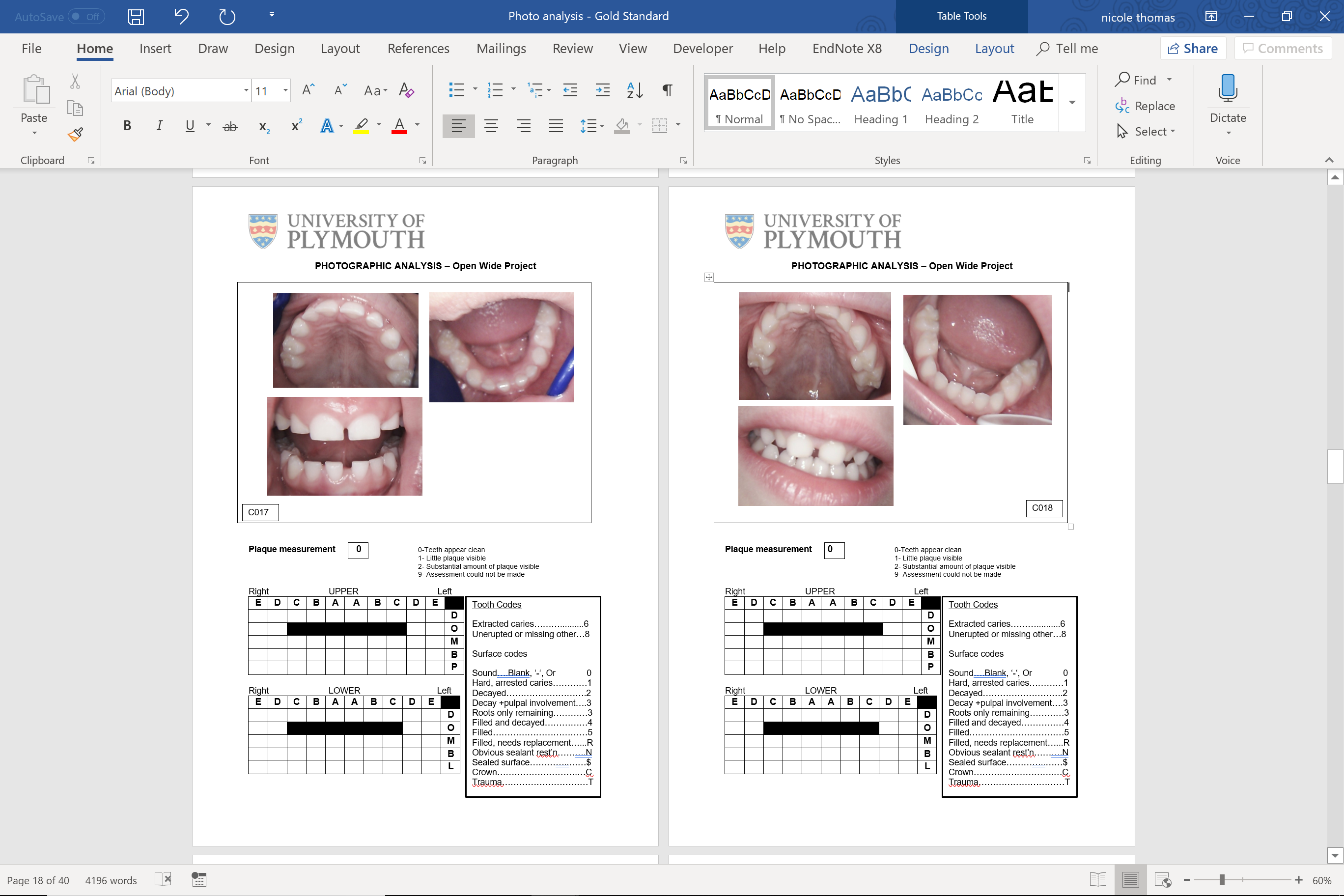


*Example of assessment sheet*

Supplement: Supplementary file 1 — Supplementary information [file 41405_2021_87_MOESM1_ESM.docx]
